# Effect of a smartphone self-management digital support system for low-back pain (selfBACK) among workers with high physical work demands – secondary analysis of a randomized controlled trial

**DOI:** 10.5271/sjweh.4186

**Published:** 2024-12-01

**Authors:** Charlotte Diana Nørregaard Rasmussen, Louise Fleng Sandal, Andreas Holtermann, Mette Jensen Stochkendahl, Paul Jarle Mork, Karen Søgaard

**Affiliations:** 1 The National Research Centre for the Working Environment, Copenhagen, Denmark.; 2 Department of Sports Science and Clinical Biomechanics, University of Southern Denmark, Odense, Denmark.; 3 Department of Public Health and Nursing, Norwegian University of Science and Technology (NTNU), Trondheim, Norway.

**Keywords:** AI, app, artificial intelligence, musculoskeletal pain, workplace intervention

## Abstract

**Objective:**

This study aimed to investigate whether physical work demands modify the effect of the selfBACK app, which is designed to support self-management of low-back pain.

**Methods:**

In a secondary analysis of the selfBACK trial with 346 employed participants, we stratified into low (N=165) and high physical work demands (N=181). Outcomes included the Roland-Morris Disability Questionnaire (0–24), a numeric rating scale for low-back pain intensity (0–10), the Pain Self-Efficacy Questionnaire (0–60), and work ability (0–10). Intervention effects were assessed at three- and nine-month follow-ups using a linear mixed model.

**Results:**

At three months, high physical demand workers with selfBACK showed a significant reduction in pain intensity [-0.8, 95% confidence interval (CI) -1.3– -0.2] compared to usual care. By nine months, the high physical demands workers with selfBACK reported reduced pain-related disability (-1.4, 95% CI -2.7– -0.1), improved pain self-efficacy (3.5, 95% CI 0.9–6.0), and lower pain intensity (-1.0, 95% CI -1.6– -0.4) compared to usual care. Low physical demands workers with selfBACK also improved pain self-efficacy [2.8 (95% CI 0.3–5.3)] compared to usual care. The impact of selfBACK was more noticeable among workers with high physical demands compared to their low physical demand counterparts, but no statistically significant differences were found in any outcome.

**Conclusions:**

The selfBACK intervention had consistent effects across workers with high and low physical work demands, indicating that these demands did not modify its impact. Both groups experienced similar positive effects, highlighting the intervention’s effectiveness across varying levels of physical work demands.

Low-back pain is among the most common health issues globally and is the world-leading cause of years lived with disability (1, 2). The burden of low-back pain imposes considerable consequences and costs for workplaces and society due to sickness absence, reduced productivity, and premature dropout from the workforce (1, 2). In Denmark, with a population of 5.9 million, the annual cost of low-back pain attributed to productivity loss (sickness absence and early retirement) is at least €2.8 billion (3, 4).

Low-back pain is especially a problem among workers with physically demanding work (ie, work that involves lifting, pulling, pushing, forward bending, and awkward postures). While some workers can work despite low-back pain, it may for others lead to poor work ability, sick leave, and premature exit from the labor market (5–7). Advice to “stay physically active (as tolerated)” plays a key role in the management of low-back pain (8–12). However, what physical activity constitutes is highly dependent on the type of job of people. It has been shown that blue-collar workers have high levels of occupational physical activity and are more fatigued compared to their peers in white-collar jobs (13). Thus, it can be difficult to motivate them to follow physical activity advice, and it is uncertain if increased physical activity will benefit their back pain.

Clinical guidelines for the management of low-back pain consistently endorse self-management as a core component together with exercise therapy (8–12). Self-management includes advice and education about the nature of low-back pain such as encouragement to avoid bed rest, stay active, and continue with usual activities including work (8–12). Educational (eg, cognitive behavioral training) and exercise interventions have been tested in workplace interventions where trained occupational health and safety ergonomists (14) or researchers delivered the intervention (15) and have proven to reduce low-back pain (14) and increase work participation (15). However, interventions that require ergonomists are often expensive and require extensive involvement of the organization to ensure implementation and sustainability (16–18). Therefore, there is a high need for effective low-cost solutions that can be made available for workers and organizations, and that reduces the burden of low-back pain for workers and workplaces.

The use of mHealth is a potentially viable option for delivering individually tailored self-management support that can improve work efficiency and reduce the costs of low-back pain. In 2019, we conducted a multinational randomized controlled trial of the selfBACK digital support system, which was designed to deliver evidence-based, individually tailored self-management support for people with low-back pain through an artificial intelligence (AI)-based app (19–21). The randomized controlled trial showed a small, but statistically significant reduction in low-back pain-related disability that was consistent and sustained over time (22).

selfBACK was developed to support self-management of non-specific low-back pain, without considerations to the physical work demands of the workers, (eg, whether they had mainly sedentary work or high physical work demands). An app-based intervention targeting low-back pain could potentially have different effects among workers with physically demanding work compared to sedentary workers. Since physical activity and exercise are two of the main components of the selfBACK intervention, the advice may be perceived and implemented differently among workers with high vs low physical work demands. This distinction may arise because (i) physically demanding work could impact workers’ comprehension of advice (eg, performing exercises when already engaged in strenuous physical activity at work), and (ii) the demands of their work might diminish their capacity or energy for sustaining behavioral changes.

Understanding the impact of smartphone-based self-management digital support systems for low-back pain among workers with high physical work demands is crucial for optimizing cost-effective interventions for a population with significant needs. Unfortunately, high-quality research is non-existent on this topic. The main aim of this study was therefore to investigate whether physical work demands modify the effect of the selfBACK app, designed to support self-management of low-back pain.

## Methods

### Design

The present study is a secondary analysis of the selfBACK randomized controlled trial, including 346 workers employed either full- or part-time during their participation in the trial (22). The trial was registered in ClinicalTrials.gov before trial start (NCT03798288) and the trial followed a predefined and published protocol (19). The study protocol was approved by the data protection agency in Denmark (201-57-0008), the regional committees for ethics in medical research in Norway (2017/923-6), and Denmark (S-20182000-24). The study is reported following the CONSORT guideline (23). All participants gave their written informed consent before entering the trial.

### Study population

We recruited the study population through their primary care practitioner (ie, physiotherapists, chiropractors, or general practitioners) in Denmark and Norway and at an outpatient clinic in Southern Denmark (the Spine Centre) when they were seeking care for low-back pain. The healthcare professionals informed possible participants about the study and provided contact information to the research group. Eligibility criteria were; having low-back pain within the last 8 weeks, rating ≥6 on the Roland-Morris Disability Questionnaire (RMDQ), having a smartphone with sufficient operating system and a working e-mail address. Exclusion criteria were; <18 years of age, having problems understanding, speaking, or reading the national language, reporting mental or physical conditions limiting participation, being unable to exercise, having a fibromyalgia diagnosis, being pregnant, having previous back surgery or participating in other low-back pain research projects. Furthermore, for inclusion in the current secondary analysis of the randomized controlled trial, the participants had to be in employment. Employment status was based on self-reported information with the following response options: full-time work, part-time work, and the following categories being characterized as the non-working population: unemployed, retired, education/military, or not employed for other reasons.

### Physical work demands

Physical work demands were estimated by the following question by Saltin & Grimby: “Which description most precisely covers your pattern of physical activity at work?” (24). Groups were defined according to the following responses: (i) You are mainly sedentary and do not walk much around at your workplace, for example, desk work, and work including assembling of minor parts; (ii) You walk around quite a bit at your workplace but do not have to carry heavy items, eg, light industrial work, non-sedentary office work, inspection and the like; (iii) Most of the time you walk, and you often have to walk upstairs and lift various items. Examples include mail delivery and construction work; (iv) You have heavy physical work. You carry heavy burdens and carry out physically strenuous work, eg, work including digging and shoveling. In the analyses, the study population was stratified into two groups at baseline based on their physical activity: group 1 being low physical activity/sitting (item i) and group 2 being high physical activity/heavier work (items ii, iii, and iv). We examined the distribution of participants across the subcategories to determine whether to use two or more groups across physical work demands, and as the very few participants were categorized as heavy physical work demands, we found the two groups to be the best solution for a balanced participant distribution.

### Randomization and blinding

A permuted block randomization was performed using a web-based trial management tool and was stratified for the country (ie, Denmark or Norway) and recruitment site (ie, physiotherapist, chiropractor, general practitioner, Spine Centre). Blinding of participants was not possible due to the nature of the intervention.

### Intervention

*selfBACK group – intervention group.* The participants were granted usage of the selfBACK app and were directed to adhere to the guidance and treatment provided by their healthcare practitioners. In a personal meeting with a study coordinator from the research group, the participants downloaded the selfBACK app on their smartphone and synchronized the app to a step-detecting wristband (Mi Band3, Xiaomi). The participants also got a general introduction to the app. The selfBACK app provided weekly individually tailored plans for physical activity, exercise, and education and aimed to support the participant to self-manage their low-back pain. The tailoring of the weekly self-management plans was provided for each component according to individual characteristics, symptoms, and symptom progression. The tailoring was achieved by using the case-based reasoning methodology, a branch of knowledge-based AI (25). The selfBACK app also includes a toolbox with tools such as goal setting, mindfulness audios, pain-relieving exercises, and sleep reminders, as well as general educational content related to low-back pain. The participants received encouraging push notifications triggered by their self-management behavior to motivate and reinforce the desired behavior. Detailed information about the selfBACK app is published elsewhere (19–21).

*Usual care – control group.* The usual care group formed the control group of the study, and the participants were instructed to follow the advice and treatment given by their healthcare professionals and manage their low-back pain as they normally would. At the end of the intervention period, they were offered a Mi Band.

### Outcomes and measurements

All outcomes in the present study were collected with a web-based questionnaire answered at baseline and six weeks and three-, six- and nine-month follow-up. The primary outcome of the randomized controlled trial was low-back pain-related disability at three-month follow-up assessed using the RMDQ (0–24 scale) (26), which is a recommended instrument for clinical trials in non-specific low-back pain (27). Moreover, outcomes included validated questions on pain intensity assessed as average low-back pain within the past week on an 11-point numerical rating scale (NRS) ranging from 0 to 10 (28), pain self-efficacy questionnaire (PSEQ) (0–60 scale) (29) and work ability (0–10 scale) (30). In addition, demographic and descriptive data were collected in the baseline questionnaire. Data are presented for all time points, with statistical analysis presented for three- and nine-month follow-ups only.

### Data analysis

To examine effect modification, we tested for differences in the intervention and control groups across various categories of physical work demands at three and nine months. The analyses were performed following the intention to treat principle estimating mean group difference with 95% confidence intervals (CI) from a linear mixed model at three and nine months after baseline, using data from all available time points, eg, baseline, six weeks and three, six, and nine months. To account for the dependency in observations within participants over time, the linear mixed model included a random intercept for each participant. To address baseline differences, we created a variable that characterized the population by time and group allocation, omitting the group term at baseline. Consequently, the population had a common baseline, not differentiated between control and intervention groups, effectively controlling for potential between-group differences. All estimated means were adjusted for the stratification variables (country, care provider) and potentially predicting variables [age (years), sex (male, female), duration of low-back pain (1, 4, 12, >12 weeks), and average low-back pain intensity within past week (11 point NRS)]. Assumptions related to normality and homogeneity of residuals, and normality of random intercepts were assessed for all models. Statistical significance was defined as P<0.05. All analyses were performed using Stata, 209 version 17.1 (StataCorp LLC, College Station, TX, USA).

## Results

**Figure 1:**
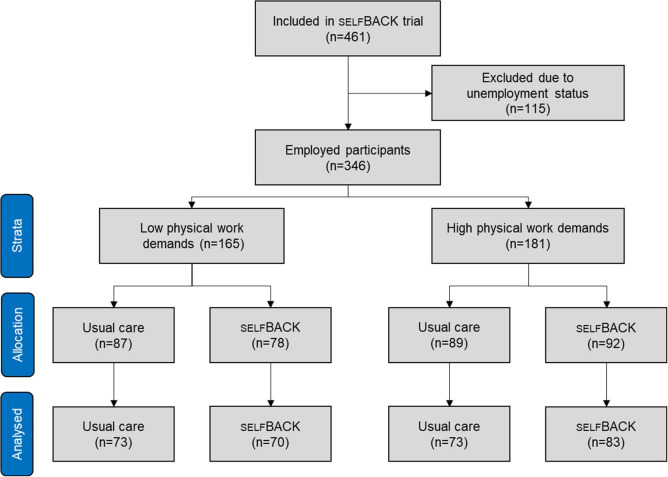
Flow over participants.

Figure 1 provides information about the participant flow. Among the 461 enrolled and randomly assigned participants to the selfBACK trial, there were 346 in employment. Of these, 165 reported to have low physical work demands, and 181 reported to have high physical work demands. Table 1 shows the baseline characteristics of the two groups. There were some differences in education with those having low physical work demands also having higher education. Additionally, those having high physical work demands also had higher intensity of low-back pain. However, other sociodemographic characteristics, back pain history, and outcome levels were similar between the two groups at baseline.

**Table 1:** Demographics and baseline characteristics for the employed population in the selfBACK trial. [SD=standard deviation; NRS=numerical rating scale]

Physical work demands		Low (N=165)					High (N=181)			
		N	%	Mean	SD (range)		N	%	Mean	SD (range)
Sociodemographic characteristics										
	Age, years,			46.1	11.3 (20-74)				43.8	12.7 (20-70)
	Body mass index, kg/m^2^			27.4	4.6				27.9	5.2
Sex, women		87	53				93	51		
Education (years)										
	0–10	2	1				18	10		
	10–12	27	16				65	36		
	>12	136	82				98	53		
Family status										
	Living alone	18	10				30	17		
	Living with other adults	57	35				69	38		
	Living with other adults and children	82	50				71	39		
	Living with children	8	5				11	6		
Low-back pain history										
	Average pain past week, NRS			4.4	1.9				5.2	1.8
	Worst pain past week, NRS			6.2	2.0				6.9	1.8
Duration of the current episode of pain (weeks)										
	<1	4	2				9	5		
	1–4	39	24				40	22		
	4–12	31	19				31	17		
	>12	91	55				101	56		
Days with low-pain past year										
	1–7	3	1				9	5		
	8–30	28	17				25	14		
	>30	67	41				78	43		
	Every day	67	41				69	38		
Use of pain medication past week (days)										
	0	25	15				35	19		
	1–2	26	16				38	21		
	3–5	46	28				49	27		
	Every day	68	41				59	33		

Table 2 shows the effect of the selfBACK intervention compared to usual care stratified by physical work demands. At three months, workers with high physical work demands receiving the selfBACK intervention demonstrated a statistically significant decrease in low-back pain intensity [adjusted mean difference ‐0.8 (95% CI ‐1.3– ‐0.2)] compared to their counterparts with similar physical work demands who received usual care.

At nine months, workers with high physical work demands receiving the selfBACK intervention reported a statistically significant reduction in low-back pain-related disability [adjusted mean difference -1.4 (95% CI -2.7– -0.1)] and an improvement in pain self-efficacy [adjusted mean difference 3.5 (95% CI 0.9–6.0)]. Furthermore, workers with high physical work demands in the selfBACK group exhibited a statistically significant decrease in low-back pain intensity at nine months [adjusted mean difference ‐1.0 (95% CI ‐1.6– ‐0.4)] compared to their counterparts receiving usual care. Workers who reported low physical work demands and who received the selfBACK intervention had statistically significant improved pain self-efficacy at nine months [adjusted mean difference 2.8 (95% CI 0.3 –5.3)] compared to workers with the same physical work demands and who received usual care. There was a tendency towards a difference in low-back pain intensity between the two groups at nine months [adjusted mean difference -0.8 (95% CI -1.6–0.1) P=0.069], with a greater reduction among workers with high physical work demands. There were no within or between sub-group differences in work ability (table 2).

**Table 2:** The effect of the selfBACK intervention compared to usual care stratified by physical work demands. [RMDQ=Roland-Morris Disability Questionnaire; PSEQ=pain self-efficacy questionnaire; SD=standard deviation; CI=confidence interval; LLM=linear mixed model]

Outcome		Low physical work demands (N=165)					High physical work demands (N=182)				Difference between low vs high work demands in the difference between usual care vs. selfBACK intervention
		Mean (SD) ^a^		Crude diff.	Adjusted ^b^ mean diff. (95% CI)		Mean (SD)^a^		Crude diff.	Adjusted ^b^ mean diff. (95% CI)	
		Usual care (N=87)	selfBACK N=78)				Usual care (N=90)	selfBACK (N=92)			
RMDQ											
	Baseline	10.1 (4.5)					10.6 (4.1)				
	6 weeks	6.4 (5.1)	6.9 (4.5)				8.4 (5.2)	7.4 (4.5)			
	3 months	6.4 (5.2)	6.4 (4.8)	-0.0	‐0.1 (-1.3–1.1)		7.7 (5.7)	6.8 (4.6)	‐0.9	‐0.9 (‐2.2–0.2)	‐0.9 (‐2.6–0.8), P=0.323
	6 months	6.2 (5.4)	5.9 (5.2)				7.1 (5.5)	5.6 (4.8)			
	9 months	6.2 (5.9)	6.3 (5.5)	0.1	-0.0 (‐1.3–1.3)		6.5 (5.1)	5.0 (4.9)	‐1.3	-1.4 (-2.7– -0.1) ^c^	‐1.4 (‐3.2–0.4), P=0.123
Average low-back pain past week											
	Baseline	4.4 (1.9)					5.2 (1.8)				
	6 weeks	3.6 (2.3)	3.3 (2.0)				4.4 (2.0)	3.5 (2.3)			
	3 months	3.6 (2.5)	3.2 (2.2)	‐0.4	‐0.4 (‐1.0–0.1)		4.0 (2.2)	3.2 (2.1)	‐0.8	‐0.8 (‐1.3– ‐0.2) ^c^	‐0.3 (‐1.1–0.5), P=0.404
	6 months	3.3 (2.4)	3.2 (2.3)				4.1 (2.2)	3.0 (2.2)			
	9 months	3.4 (2.5)	3.3 (2.4)	-0.2	‐0.2 (‐0.8–0.4)		3.8 (2.1)	2.7 (2.2)	‐0.9	‐1.0 (‐1.6– ‐0.4) ^c^	-0.8 (-1.6–0.1), P=0.069
PSEQ											
	Baseline	46.1 (10.2)					43.4 (11.3)				
	6 weeks	48.5 (11.4)	47.6 (9.8)				44.4 (12.0)	48.3 (9.3)			
	3 months	48.5 (11.4)	50.3 (9.0)	1.8	1.8 (‐0.5–4.3)		46.7 (10.4)	48.9 (9.9)	2.2	2.2 (-0.1–4.6)	0.3 (‐3.0–3,7), P=0.846
	6 months	48.6 (11.4)	50.0 (9.4)				47.6 (10.5)	50.6 (10.4)			
	9 months	47.9 (11.1)	50.6 (8.9)	2.7	2.8 (0.3–5.3) ^c^		48.0 (10.7)	51.5 (9.1)	3.5	3.5 (0.9– - 6.0) ^c^	0.7 (‐2.9–4.2), P=0.719
Work ability											
	Baseline	7.1 (2.1)					6.3 (1.9)				
	6 weeks	7.4 (2.0)	7.5 (1.5)				6.7 (1.8)	7.0 (2.1)			
	3 months	7.5 (2.0)	7.6 (1.7)	0.1	0.1 (‐0.4–0.7)		6.8 (2.1)	7.2 (1.8)	0.4	0.4 (‐0.1– 0.9)	0.2 (‐0.5–1.0), P=0.513
	6 months	7.3 (1.9)	7.3 (2.0)				6.7 (2.0)	7.3 (1.9)			
	9 months	7.7 (1.9)	7.6 (2.0)	‐0.1	‐0.1 (‐0.6–0.5)		6.9 (2.0)	7.5 (1.7)	0.6	0.5 (‐0.0–1.1)	0.6 (‐0.2–1.4), P=0.139

^a^ Marginal means from a crude linear mixed model, and SD from raw data among persons with information at specific time points. ^b^ Adjusted for stratification variables (clinician, country, pain duration (4 cat), pain intensity (continuous 11 numerical rating scale), age (continuous). ^c^ P<0.05

## Discussion

Our objective was to investigate whether physical work demands modify the effect of the selfBACK app, designed for self-management of low-back pain. In summary, there was no discernible difference in the selfBACK intervention’s effect between workers with high and low physical work demands. Thus, physical work demands do not modify the effects of the selfBACK app. While the effects did not reach statistical significance, the effects of the selfBACK intervention seemed more pronounced among workers with high physical demands than their counterparts with lower physical demands. Those with high physical demands experienced statistically significant benefits in low-back pain-related disability, pain self-efficacy, and low-back pain intensity due to the intervention, whereas workers with low physical demands only showed statistically significant benefits in low-back pain intensity.

To our knowledge, this is the first randomized controlled trial to investigate the effects of a smartphone-based self-management digital support system app to support self-management of low-back pain among workers with low and high physical work demands. Previously app-based workplace interventions have targeted health promotion (31), physical activity (32, 33), work stress and well-being (34, 35), and depression (36) with varying effects. A previous mobile-Web intervention named “FitBack” targeted adults with non-specific low-back pain in various work sectors (trucking, manufacturing, technology, and corporate headquarters). FitBack incorporated tailored content, such as text articles and videos segmented to address job-specific issues and self-care activities, categorizing users by job types like sitters, standers, drivers, and lifters (37). The positive outcomes observed among both workers with low and high physical demands underpin the significance of the selfBACK intervention. While FitBack users exhibited no effect at the 2-month follow-up, a significant reduction in low-back pain emerged at the four-month mark compared to a control group without additional care. Unfortunately, subgroup analyses based on varying levels of physical work demands were not conducted in the study (37).

Although the reductions in pain-related disability and pain intensity with the SelfBACK app were modest, they align with outcomes from more resource-intensive interventions. A recent meta-analysis of workplace exercise programs reported pain intensity reductions of -0.23– -0.73 (38). Our findings of -1.0 in pain intensity and -1.4 in pain-related disability demonstrate that a low-cost and scalable app like SelfBACK can achieve comparable results to exercise programs. This underscores the app’s potential as an effective, accessible tool for managing pain in working populations.

The observed positive effects of the selfBACK intervention may be attributed to its comprehensive nature, incorporating diverse self-management strategies applicable to a range of work demands. Previous workplace interventions, integrating educational components such as cognitive behavioral training and exercise interventions, have demonstrated effectiveness in reducing low-back pain (14) and enhancing work participation (15). This suggests the relevance of selfBACK's content for workers with varying levels of work demands despite selfBACK not being explicitly tailored to specific physical work demands. The users of FitBack reported enhancements in work-related outcomes, worker productivity, and presenteeism (37). In contrast, our study did not identify improvements in work-related outcomes, specifically work ability. The absence of targeted content in selfBACK addressing job-specific issues and self-care activities related to work may explain this lack of impact on work-related outcomes. This highlights the potential importance of incorporating such elements for a positive effect on work-related outcomes.

The positive results of the selfBACK intervention hold significant importance as it points to the potential of a low-cost intervention, such as a digital app, to effectively reach and benefit a group in need. Specifically, those with high physical work demands often have lower educational levels and are situated in a lower socioeconomic group (39). Our data further supports this observation, and this high-risk group encounters distinct challenges in effectively managing pain while maintaining active employment. This issue poses a substantial burden on both society and workplaces (3, 40). Given that many workplace interventions struggle to reach individuals in lower socioeconomic groups, addressing this challenge becomes paramount. Thus, the potential of a digital, low-cost solution holds great promise, offering a significant opportunity to effectively reach and support high-risk groups.

### Perspectives

Addressing the critical need for interventions among workers with high physical work demands underscores the potential impact of tools like the selfBACK app. The demand for tailored solutions is particularly pronounced in small and medium-sized enterprises, where unique challenges in geographical dispersion and limited resources persist. The implementation and scale-up of such interventions require careful consideration of user engagement, adaptability to varying work demands, and solutions for logistical challenges. Moreover, a shift towards tailored interventions specifically designed for workplace settings, accommodating different physical work demands, is essential. Research initiatives focused on rigorous testing and implementation within workplace contexts will be pivotal in ensuring the effectiveness, feasibility, and sustainability of these interventions, ultimately catering to the diverse health needs of the working population.

Further development and evaluation of the selfBACK app are essential to target specific worker characteristics and challenges, including more specific information on adapting work to pain status and functional limitations. With respect to the role of the work environment system at the workplace, considering solutions allowing users and workplace representatives to share app content and monitor progress is important. Responding to experiences from the selfBACK trial, the development of a clinician dashboard for co-decision-making is underway (41), and a work environment dashboard should also be provided as a tool for the workplace occupational health and safety system.

### Strengths and limitations

This study has several strengths, including its randomized design and the utilization of web-based questionnaires for repeated outcome measurements. Furthermore, the randomized controlled trial adhered to the protocol and demonstrated a smaller loss to follow-up than initially anticipated.

However, some limitations should be considered when interpreting the results. Firstly, participants were not blinded, although those in the intervention group received no additional attention beyond the guidance on app installation. Secondly, the subgroup analysis was not pre-planned, and despite a relatively large sample size in the main randomized controlled trial, the study lacked the statistical power for robust secondary analyses, which may be why we don’t find effect modification. Furthermore, not all relevant workplace outcomes, such as productivity and sick leave, were assessed. The definition of physical work demands relied on self-reported information. Due to the low prevalence of participants with heavy physical work (group iv), we dichotomized data to compare participants with sedentary work to those with physical work. We cannot rule out that we have undere- or overestimated the impact of physically demanding work or that a change in cut point would have altered our conclusions. Lastly, the two groups of workers with low versus high physical job demands differ with respect to their educational level and may also differ with respect to the unobserved characteristics. However, previous secondary analysis of the selfBACK trial found no effect modification from educational level, age, or gender (42). This suggests that the observed differences in the intervention effects are more likely attributable to physical work demands rather than educational background or other characteristics.

### Concluding remarks

Overall, the selfBACK intervention had consistent effects across workers with high and low physical work demands, indicating that these demands did not modify its impact. Both groups experienced similar positive effects, highlighting the intervention’s effectiveness across varying levels of physical work demands. To enhance its effectiveness, refining and assessing the selfBACK app could tailor its features to better address specific worker and workplace characteristics and challenges. This refinement has the potential to transform selfBACK into a highly relevant and cost-effective tool, particularly beneficial for assisting workers engaged in physically demanding occupations in effectively managing back pain and maintaining productivity at work.
